# Ranking threats to biodiversity and why it doesn’t matter

**DOI:** 10.1038/s41467-022-30339-y

**Published:** 2022-05-16

**Authors:** Céline Bellard, Clara Marino, Franck Courchamp

**Affiliations:** Université Paris-Saclay, CNRS, AgroParisTech, Ecologie Systématique Evolution, 91405 Orsay, France

**Keywords:** Biodiversity, Macroecology

## Abstract

Several rankings of the relative importance of global threats to biodiversity have been proposed. This Comment argues that relative rankings of biodiversity threats have little application for conservation and might even mislead policymaking.

## Teaser

Conservation scientists have proposed several rankings of the relative importance of global threats to biodiversity. Here, we argue that relative ranking of biodiversity threats depends on local context and metrics used, and so has little application for conservation.

We are in an unprecedented crisis of biodiversity in human history. All evidence suggests that the current rates of extinctions vastly exceed the estimated background extinction rate. The major difference with the previous mass extinctions is that humans are simultaneously responsible for it, threatened by it, and able to stop it. More than 900 species across all taxa have been documented to go extinct since 1500, with probably as many as 400 bird species alone in prehistoric times^1^. For the scientific community, five main global threats are typically considered responsible of these losses: habitat destruction, over-exploitation, biological invasions, climate change, and pollution^2^, although many more local perturbations and stressors are also important.

Many individual researchers and agencies such as the Intergovernmental Science-Policy Platform on Biodiversity and Ecosystem Services (IPBES), International Union for Conservation of Nature (IUCN), and World Wide Fund for Nature (WWF) have recently ranked these global-threat categories in terms of their estimated contribution to biodiversity loss. For instance, biological invasions are deemed the primary cause of global species’ extinctions for birds, mammals, reptiles, freshwater fish, plants, arthropods, and gastropods, especially on islands^3^. Currently, more than 38,500 species are considered threatened with extinction, first by habitat loss and then overexploitation^4^. Recently, the IPBES published a ranking of these threats, with habitat change identified the most important threat, followed by overexploitation, climate change, pollution, and biological invasions^5^. Consequently, the global exercise of ranking threats at a global scale, although a natural tendency for scientists, has led to differing rankings. However, such a variability can misguide conservation responses depending on which ranking system is favored.

## Threat rank is context-specific

**Fig. 1:**
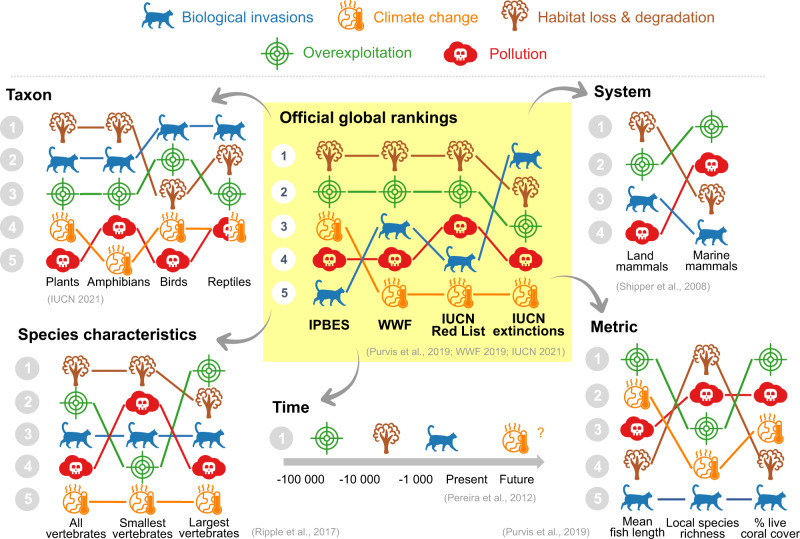
Divergence of global threat rankings across different references and international agencies. IPBES, WWF, and IUCN established global rankings of the five threats responsible for the current biodiversity crisis (B: central, yellow panel). However, the relative importance of each threat depends on the taxon, system, species’ characteristics, time, and/or the metric considered, resulting in divergences. Global biodiversity threats are represented by colors and symbols, given in the top panel. This figure encapsulates results combined from different studies detailed in Supplementary Table 1 with their associated references.

The difficulties inherent in ranking global threats are due to them being context-dependent, which result from conditions and the nature of the threats themselves differing among locations, habitats, and taxa (Fig. 1). Current high-risk hotspots from habitat loss and overexploitation are primarily located in the tropics, whereas Europe is documented as a threat hotspot for pollution^6^. On islands, biological invasions mainly threaten biodiversity in the Pacific and Atlantic Oceans, while islands in the Indian Ocean and near the coasts of Asia are mostly threatened by overexploitation and agriculture^3^. Climate change affects species more at higher latitudes and altitudes because species are constrained by the physical environment (geographic barriers and mountain tops) to follow their optimal isotherms.

The relative importance of threats also depends on the taxon considered. At the global scale, vertebrates are primarily threatened by habitat loss, overexploitation, and then biological invasions. But even within the vertebrates rankings differ — birds and mammals are mainly affected by overexploitation, while amphibians have a higher probability of succumbing to habitat loss^6^. Because of species-specific traits and adaptations, some species are likely to respond differently to global threats even within a clade. Large-bodied vertebrates are more likely to be threatened by overexploitation, whereas small-bodied vertebrates are more prone to habitat loss or pollution (Fig. 1). Threat ranking also depends on the habitat under consideration. Marine mammals are more threatened by overexploitation and pollution than terrestrial mammals for which habitat loss is the primary threat (Fig. 1). On islands, habitat loss is secondary to the pressures of biological invasions in freshwater systems, but the former is more important for terrestrial vertebrates and plants^3^. Another source of uncertainty is that most studies examining threats are based on well-studied taxa such as terrestrial vertebrates, which only represent a small subset of the tree of life. For instance, only 0.2% of fungi, 1.7% of invertebrates, and 10% of described plants are assessed in the IUCN update of 2019^7^, potentially underestimating the intensity of some threats and biasing conservation priorities for these groups. Similarly, there is a bias of research effort towards regions with high-income countries, while research from low or middle-income countries is generally underrepresented^8^. This may give the false impression of absence of threats in some regions of the world.

Likewise, period-specific global threat ranks are subject to the vagaries of temporal dynamics (Fig. 1). However, distinguishing past, current, and future threats is essential for current or future conservation interventions. Historically, overexploitation caused most of the Pleistocene megafauna extinctions, likely exacerbated by climate change. As agricultural practices intensified, habitat loss played a major role in extinctions. As humans later colonized islands, biological invasions caused the extinction of hundreds of species worldwide^3^. In contrast, climate change is only predicted to become major in the near future^9^. In fact, the effects of recent threats might be masked by delayed species’ responses, especially in under-studied regions, resulting in a large extinction debt. For instance, the severity of biological invasions often causes native species to decline rapidly to local extinction, while other threats such as habitat loss might affect species more slowly. In both cases, the eventual extinctions are ultimately if similar magnitude.

## Threat rank depends on the metric

The inconsistencies among different rankings also arise from the different methodologies applied and metrics used to assess biodiversity losses. The IUCN Classification Threat, one of the most used tools in ecology and conservation, is based on expert assessment and a list of quantitative criteria (i.e., population sizes and area of occupancy) to provide a species’ extinction probability. Adopted by the Convention of Biological Diversity (CBD), the WWF Living Planet Index assesses threats to biodiversity based on population time series of over 4000 vertebrate species. Recently, a new metric based on species’ extinction risk status, anthropogenic pressure, and country-scale mitigation measures has provided an interesting, complementary approach of congruence among species’ extinction risks and their exposure to threats^10^. However, most of the aforementioned metrics rely on species-level data that are often unavailable. Yet, because biodiversity is a multidimensional concept, including genetic, functional, and taxonomic diversities, it is important for threat assessments to consider all dimensions. Attempting to be more integrative, the IPBES proposed a new ranking based on eleven different metrics, from species populations (e.g., Living Planet Index, local species richness) and traits (e.g., mean body length) to ecosystem structure (e.g., mangrove forest, percentage of live coral cover)^5^. Those multiple metrics result in different rankings according to the scale considered. For instance, habitat loss and degradation appear to be important for explaining changes at the population level (i.e., Living Planet and Red List indices), while pollution is among the most important threats of changes of ecosystem structure (e.g., % live coral cover) (Fig. 1). Some of these metrics are less appropriate to emphasize certain threats. For example, species traits like mean body length of fish is mostly driven by overexploitation^11^, while biological invasion had little effect on this trait, or on the percentage of live coral cover. Moreover, each threat is in fact multifaceted. For instance, climate change includes sea-level rise, altered precipitation, increase of extreme events, and some of which may even have opposite effects on certain species. For example, some plants may benefit from CO2 increases while suffering from changed in precipitation regime. As a consequence, the definition of threat itself may lead to various rankings.

## Conservation implications of ranking threats

The tendency to rank biodiversity threats is not entirely due to the tendency of scientists to ordinate factors or to their relevance to justify studies of the greatest threats. It is also driven by practical considerations. Rankings are in fact commonly applied to establish priorities for conservation interventions, especially by policy makers^12^. For instance, comparative studies have successfully prioritized the conservation of some populations, species, or sites at local scales^12^. Global analyses and rankings can also guide governments and other actors, and offer leverage to implement measures with limited funding. By ranking individual threats independently, scientists implicitly prioritize the top threats and classify others as lower concern. However, ranking global threats by considering each threat independently is likely to underestimate the full consequences to biodiversity worldwide. A recent study showed that the nature of the threat is almost irrelevant when species are exposed to multiple threats simultaneously, as multiple threats exacerbate the loss of resilience in vertebrate populations^13^. Threatened insular species are currently exposed to an average of 2.6 different threats^3^, and according to the IUCN 80% of species are exposed to more than one threat threatened species^4^. In this context, we expect cumulative effects among global change drivers, which may result in unexpected interactions such as synergies or antagonistic interactions. This further weakening the usefulness of independent ranking.

## Perspectives and potential opportunities

By over-synthesizing the relative prevalence of threats across juxtaposed contexts, taxa, times, and metrics, these macroecological analyses confuse messages to the public, the media, and policy makers in terms of where and when biodiversity losses occur, and which factors are responsible^14^. Instead, global assessments in conservation should offer synthetic views on how threats operate in combination to threaten biodiversity. New developments in databases and tools could provide advances in conservation evaluation^15^. For instance, network analyses^3^ or cumulative-impact approaches^16^ can consider multiple threats simultaneously. But exposure to threats alone provides only partial information on conservation needs. New approaches assessing species’ vulnerability to global changes by simultaneously considering exposure to the intensity of the threats, variable sensitivity, and different adaptive capacity are also a valuable change of paradigm^17^.

Because the ultimate objective is to lessen the severity of the biodiversity crisis driven by combination of major threats, we might even need to reconsider the actual usefulness of global threat ranks. Ranking the potential benefits of conservation actions would certainly have more impact that only ranking threats. Providing policy makers with a range of conservation scenarios with their predicted biodiversity outcomes, which consider the multiple sources of stress, different metrics, and variation across taxa, could provide a more realistic description of the state of biodiversity and maximize conservation outcomes. While international biodiversity-conservation instruments derived by the CBD, IPBES, IUCN, and others are exceptional tools to monitor and assess biodiversity, the disparities among rankings create a reductive and oversimplified perspective of the multiple threats underlying the current biodiversity crisis. This could also precipitate unsubstantiated priorities in conservation. Although useful to highlight areas of concern, we argue instead for considering the specificities, complexities, and interactions among threats to tackle this global crisis and for communicating more strategically with policymakers about the limits of global rankings.

## Supplementary information

Supplementary Information
