# Supplementary material for: Ranking threats to biodiversity and why it doesn’t matter
Source: Nat Commun. 2022 May 16;13:2616. doi: 10.1038/s41467-022-30339-y (PMC9110410; doi:10.1038/s41467-022-30339-y)
Supplement: Supplementary file 1 — Supplementary Information [file 41467_2022_30339_MOESM1_ESM.pdf]

Supplementary Information for **Ranking threats to biodiversity and why it doesn't matter**

Supplementary Table 1
Supplementary references (1–6)

Supplementary Table 1.

Panel	Sub-panel	Metrics	Ranking of the threats					Reference
			BI	CC	HL	O	P	
**Official global rankings**	IPBES	Relative impacts of threats on biodiversity at a global scale combining different metrics operating at different scales (e.g., species population, species traits, community composition, ecosystem function, and ecosystem structure, see supplementary material of IPBES Global assessment Chapter 2.2 for details)	5	3	1	2	4	[1]
	IUCN Red List	The most prevalent threats for threatened or near-threatened species from comprehensively assessed species groups N = 25600 species	4	5	1	2	3	[1]
	IUCN extinctions	Number of species extinctions associated to each threat (all taxa assessed by the IUCN) N = 122500 species (including 976 extinct)	1	5	2	3	4	[2]
	WWF	Average rate of population change across time series of vertebrate species N = 27000 populations of 4300 vertebrates	3	5	1	2	4	[3]
**Taxon**	Plants	Number of species extinctions associated to each threat using the IUCN Red List N = 79500 species	2	4	1	3	5	[2]
	Amphibians		2	5	1	3	4	
	Birds		1	4	3	2	5	
	Reptiles		1	4°	2	3	4°	
**Species characteristics**	All vertebrates	Number of species facing each threat related to their body size N = 27600 vertebrates	3	5	1	2	4	[4]
	Smallest vertebrates		3	5	1	4	2	

	Largest vertebrates		3	5	2	1	4	
**Time**	-100,000 to -10,000 y	Major threat associated to species extinctions through time based on the literature	/	/	/	1	/	5
	-10,000 to -1,000 y		/	/	1	/	/	
	-1,000 y to present days		1	/	/	/	/	
	Future		/	1	/	/	/	
**Metric**	Mean fish length	Change in mean fish length over time	5	2	4	1	3	1
	Local species richness	Change in number of species within communities over time	5	4	1	3	2	
	% live coral cover	Modelled global average in coral cover trend across time	5	3	4	1	2	
**System**	Land mammals	Number of species affected by each threat	3	NC	1	2	4	6
	Marine mammals		4	NC	3	1	2	

Threat rankings and associated definition. This table contains the rankings extracted from different sources (Reference column) that were used to realized Fig. 1. The Panel column refers to the different sections of Fig. 1, Sub-panel refers to the different levels within a panel. We provide the definition of each indicator corresponding to the various rankings and the number of species or population assessed for its calculation if applicable (Metric column). Threats (BI = biological invasions, CC = climate change, HLD = habitat loss and degradation*, O = overexploitation[†], P = pollution) are ranked from 1 the most important to 5 the least important. NC = not considered.

° Climate change and pollution lead to the exact same number of extinctions for reptiles (n = 9 species). They are both the 4th threat associated to reptile extinctions.

* Habitat loss and degradation is a global driver that is composed by many processes. For instance, in the IUCN Threat Classification Scheme, six threat categories contribute to habitat loss: residential and commercial development, agriculture and aquaculture, energy production and mining, transportation and service corridors, human intrusion and disturbance, natural system modification. We thus aggregated all these categories to result in the global driver

“Habitat loss and degradation” for all metrics based on the IUCN Red List, or that used the same threat classification (e.g. Ripple et al, 2017).

†Overexploitation corresponded to biological resource use. For land and marine mammals, Shipper et al (2008) evaluated also the threat “accidental mortality” that was the first threat associated to marine mammals. As this threat is directly due to fishing in open seas and accidental catch of mammals, we pooled it with overexploitation of biological resources.

Supplementary references:

1. Purvis, A. *et al. Chapter 2.2. Status and Trends – Nature. In: Global assessment report of the Intergovernmental Science-Policy Platform on Biodiversity and Ecosystem Services*. (2019).
2. IUCN. The IUCN Red List of Threatened Species. Version 2020-3. (2020). Available at: IUCN 2020. %0A(downloaded on January 2022)%0A. (Accessed: 15th January 2022)
3. WWF, Almond, R.E.A., G. M. and P. *Living Planet Report 2020 - Bending the curve of biodiversity loss*. (2020).
4. Ripple, W. J. *et al.* Extinction risk is most acute for the world’s largest and smallest vertebrates. *Proc. Natl. Acad. Sci.* **114**, 10678 LP – 10683 (2017).
5. Pereira, H. M., Navarro, L. M. & Martins, I. S. Global Biodiversity Change: The Bad, the Good, and the Unknown. *Annu. Rev. Environ. Resour.* **37**, 25–50 (2012).
6. Schipper, J. *et al.* The status of the world’s land and marine mammals: diversity, threat, and knowledge. *Science (80).* **322**, 225–230 (2008).
